# Towards an Equitable Digital Society: Artificial Intelligence (AI) and Corporate Digital Responsibility (CDR)

**DOI:** 10.1007/s12115-021-00594-8

**Published:** 2021-06-14

**Authors:** Karen Elliott, Rob Price, Patricia Shaw, Tasos Spiliotopoulos, Magdalene Ng, Kovila Coopamootoo, Aad van Moorsel

**Affiliations:** 1 School of Computing & Business School, Newcastle University, 1 Science Square, The Helix, Newcastle upon Tyne, NE4 5TG UK; 2 http://CorporateDigitalResponsibility.net (CDR), Alchemmy, 52-54 High Holborn, London, WC1V 6RL UK; 3 Beyond Reach Consulting Ltd, 139 Furlong Road, Bolton-Upon-Dearne, Rotherham, S63 8HD UK

**Keywords:** Artificial intelligence (AI) governance, Digital ethics and trust, Complexity, Corporate Digital Responsibility, Equitable digital society, Financial technology (FinTech)

## Abstract

In the digital era, we witness the increasing use of artificial intelligence (AI) to solve problems, while improving productivity and efficiency. Yet, inevitably costs are involved with delegating power to algorithmically based systems, some of whose workings are opaque and unobservable and thus termed the “black box”. Central to understanding the “black box” is to acknowledge that the algorithm is not mendaciously undertaking this action; it is simply using the recombination afforded to scaled computable machine learning algorithms. But an algorithm with arbitrary precision can easily reconstruct those characteristics and make life-changing decisions, particularly in financial services (credit scoring, risk assessment, etc.), and it could be difficult to reconstruct, if this was done in a fair manner reflecting the values of society. If we permit AI to make life-changing decisions, what are the opportunity costs, data trade-offs, and implications for social, economic, technical, legal, and environmental systems? We find that over 160 ethical AI principles exist, advocating organisations to act responsibly to avoid causing digital societal harms. This maelstrom of guidance, none of which is compulsory, serves to confuse, as opposed to guide. We need to think carefully about how we implement these algorithms, the delegation of decisions and data usage, in the absence of human oversight and AI governance. The paper seeks to harmonise and align approaches, illustrating the opportunities and threats of AI, while raising awareness of Corporate Digital Responsibility (CDR) as a potential collaborative mechanism to demystify governance complexity and to establish an equitable digital society.

## Introduction

The global financial crisis (GFC, 2007–2009) marked a significant failure in citizens’ trust and questioned governance mechanisms in financial services (Pedersen 2021). Several algorithms ceased working during the crisis because of the degree of stress and strain on the calibration designed in a different epoch, a calibration that lacked the ability to adapt the behaviour of the algorithm under a crisis situation, creating a breakdown of societal trust in artificial intelligence (AI) (Edelman 2019a, 2019b). Such concerns are revealed in public perceptions and uncertainty surrounding AI’s future in society from technology executives overseeing the development and implementation of AI to the general public (in the USA). Key findings underpin the central tenets of the field of “AI and Society”, which this paper examines. On the one hand, industry and people are curious about “good” AI—the opportunities and benefits. On the other hand, we observe substantial uncertainty and anxiety that the rapid adoption of AI across the digital “space” will impact society in negative ways: widespread job loss, income inequality, and social isolation (ibid.). How can citizens be included and benefit from AI innovations and, specifically, protect their data and digital identities in the fourth industrial revolution (Kowalikova et al. 2020; Lewis 2021; Hamdan et al. 2021)? And, where does accountable responsibility rest to ensure that we establish an equitable digital society for all, and not the few? To place the debate on the associated risks of AI and societal implications in context, first, we review pertinent statistical insights in this area; second, we provide a brief background to our current financial services (FS) and financial technology (FinTech) environment, specifically, AI/FinTech-enabled FS; third, we examine trust, ethical principles, and regulatory implications (including the new EU guidance). Last, we introduce Corporate Digital Responsibility (CDR) as a potential collaborative mechanism to navigate such complexity, proposing guidance frameworks towards responsible corporate digital actions in preserving societal interests.

## AI Perceptions

Edelman’s AI survey (2019b) findings raise fundamental questions for AI/FinTech-enabled FS adoption and associated societal implications. The public reported slightly higher AI concerns than the tech executives canvassed in the USA (percentages indicated respectively); *safe development—*60/54%; *the roles of society*, *business*, *and government—*91/84%; *hurting the poor—*54/43%; *benefiting the wealthy—*67/75%; *loss of human intellectual capabilities—*71/65%; *increased social isolation—*74/72%; *society feels threatened—*81/77%; and *highly corrosive to public trust—*51/45%. Each finding highlights the interplay between perceived AI risks, opportunities, and threats that are interwoven within our discussion surrounding notions of trust, ethics, legality, and governance nested in societal systems and people’s relationship with AI. Simply put, technologies have permeated social interaction whether on digital platforms or the positives afforded by Zoom during the recent pandemic (Wiederhold 2020; Haochen and Polak 2021). Online interactions are imbued with decisions around our levels of awareness regarding privacy and security of our data, in readily accepting the “T&Cs” (terms and conditions), or General Data Protection Regulation (GDPR 2018) compliance via the click of a digital button to access information we desire. The technology that lies beneath, regulating and accessing a range of complex systems, that can arbitrarily create a characteristic profile of our digital “selves” and AI continues to develop.

## AI Development

Lepore (2020: 2) names the Simulmatics Corporation as first to draw on Turing’s insights and “engage principally in estimating probable human behaviour by the use of computer technology.” Simulmatics is “[t]he long-dead grandparents of the data-mad, algorithmic twenty-first century” (ibid: 4-5). This corporation employed the “What-If Men” who forged ahead on the assumption that in replicating human behaviour, many societal disasters and risks could be averted via the use of technology. As a result, this group instigated the “future computed” as AI now dominates society’s symbiotic existence (Microsoft 2018). Moving forward, Cybenko’s (1989) “Universal approximation theorem” proof showed that artificial neural networks (ANNs) can, with arbitrary precision, approximate all continuous functions with a finite number of learning nodes. This is a powerful feature of AI-based algorithms. For instance, distributed ledger technology (DLT) that emerged in 2008 employs this powerful feature, providing multiparty computations wide-scale transaction clearing with mutual distrustful parties, termed FinTech, opening a universe of AI/FinTech-enabled financial services (FS). A recent use-case leveraging DLTs/AI/FinTech-enabled FS is open banking (OB). Customers are given access to their own data, previously the reserve of traditional financial institutions and data services “records of everything upon which a customer makes an electronic payment”, permitting products and services tailored to the fluctuating financial habits and demands of the tech “savvy” customer (Bell 2020: 183). As we shall reveal, a feature of AI-governance is that rather than “compliance” with AI regulation or statutes of law deemed necessary, “doing good business” becomes a cultural “norm” exemplified within OB practices to benefit society (Durodié 2019: 121).

This overview demonstrates AI’s potential for “good” societal interventions—from increased efficiency in analysing large datasets, reduction of mundane tasks with reliability and consistency, opening financial services, to complementing hazardous human tasks—bomb disposal, and precise medical interventions (Maddeo and Floridi 2018). Given the “good” tempered with the caveat of “bad” public opinion in the Edelman (2019a, 2019b) survey, and the reported mistrust of AI systems to behave in accordance with its intended purpose, do we know that AI engineering decisions are unbiased and fair and promote equality of use aligned with societal values? (Aitken et al. 2020). A significant issue surrounds the “black-box” element of AI whereby computer scientists and engineers find that the machine learning systems used for predicting an outcome remain opaque or cannot be adequately observed. Granted, a level of acceptance can be discerned to satisfy machine learning principles, but progress in trying to resolve these issues will be a feature of auditability and understanding of AI decisions ex post to enhance broader uptake of machine learning and thus, AI (Pasquale 2015, 2017). Explaining the internal mechanics of the deep or machine learning system in human terms or explainable AI (XAI) remains problematic because there is “[n]o real consensus about what interpretability is in machine learning” (Molnar 2019: 31). Hence, the XAI body of literature is nascent and may take several years to explore, understand, and translate into a useable format (Elton 2020). Meanwhile, regulators and the law attempt to address the risks and culpability of the “black box” phenomenon of AI systems, decisions, and subsequent societal effects (Pasquale 2015, 2017). What is required is to move “from more AI to better AI” (Pasquale 2020: 196).

At the time of writing, nothing is compulsory in terms of regulation, AI governance, ethics, and legal compliance trail the rate of AI innovation and implementation in digital society (Floridi 2019; Roitblat 2020). Indeed, as Mittelstadt (2019: 501) asserts, “ethical principles are not enough.” Certain flaws exist in the “84 public-private initiatives” he explored regarding adequately defined duties, accountability, linkage to societal norms, and a framework of methods for AI implementation and governance. Over 160 ethical principles exist to date, suggesting fragmentation and difficulty in choosing which principles are best for responsible use of AI (AlgorithmWatch 2020). The European Union consulted across academia, industry, and policymakers to agree upon a set of co-created standards for AI Regulation—released on 21 April 2021 (EU, 2021)^1^. Yet, early insights indicate that interpretation of the EU rules, compliance, and enforcement are “vague”, and “loopholes” are already identified in the draft documentation (Bloomberg 2021; The Verge 2021); we will return to this regulation later. Now, we move to examine the digital society landscape and its social actors.

## Digital Society

Zuboff (2019) examined digital society, AI, and symbiotic social relationships, claiming that society is subject to *Surveillance Capitalism*. Technologies track our every movement, and almost unaware, we have slipped into accepting digital surveillance as a daily norm. Despite adopting a predominant Western perspective, Zuboff’s (2019: 199-232) work draws attention to the discourse we unpack; she reveals the notion of overt and covert data manipulation, whereby users have a “puppet and puppet master” relationship (ibid.: 14-17)—the puppet representing a device to grant access to the digital society, which has AI systems operating in the background, harvesting data insights and forming arbitrary user profiles aligned to the masters’ interests (cf. Cybenko 1989). She argues this process is enabled via the “forward text” and the “shadow text”. The former is the user experience (UX), to be aware of our data and who is using said data, when engaged online via social media platforms, i.e. Google and Facebook. UX is premised on an array of alluring features to “hook” and retain user’s attention and stimulate their desire for continued engagement. Conversely, the “shadow” text, as the term suggests, describes a covert system owned and manipulated to benefit the master, via sharing and monetising user’s data with selected third parties. Transparency to the user and society is blurred, for the tech giants operate largely without regulation, using and re-using data (ibid.). A moot point is the exploitation of adolescence, “[y]oung life now unfolds in the spaces of private capital, owned, and operated by surveillance capitalists…operationalised in practices designed to maximise surveillance revenues” (Zuboff 2019: 456). Adulthood mirrors this description, as the puppet master lures individuals to join the digital society (ibid.: 335):

the machine intelligence capabilities to which data are continuously supplied, the analytics that discern patterns, and the algorithms that overt them into rules . . . the essence of the uncontract, which transforms the human, legal and economic risks of contracts into plans constructed, monitored and maintained by private firms for the sake of guaranteed outcomes: less contract utopia than *uncontract dystopia*

Translating the uncontract for AI/FinTech-enabled FS, we suggest that beyond OB, society has been changed, premised on the new “normative judgment”, that the digital society is a better state than the prior (Byrne 2010: 62). Byrne (ibid.) draws our attention to the pivotal ethical question involving the normative self and how the plethora of actors within AI/FinTech-enabled FS interact: we “assume that “we” represent some sort of universal interest—in reality there are often conflicting interests at play—what works for whom?”

## Vested Interests

**Fig. 1:**
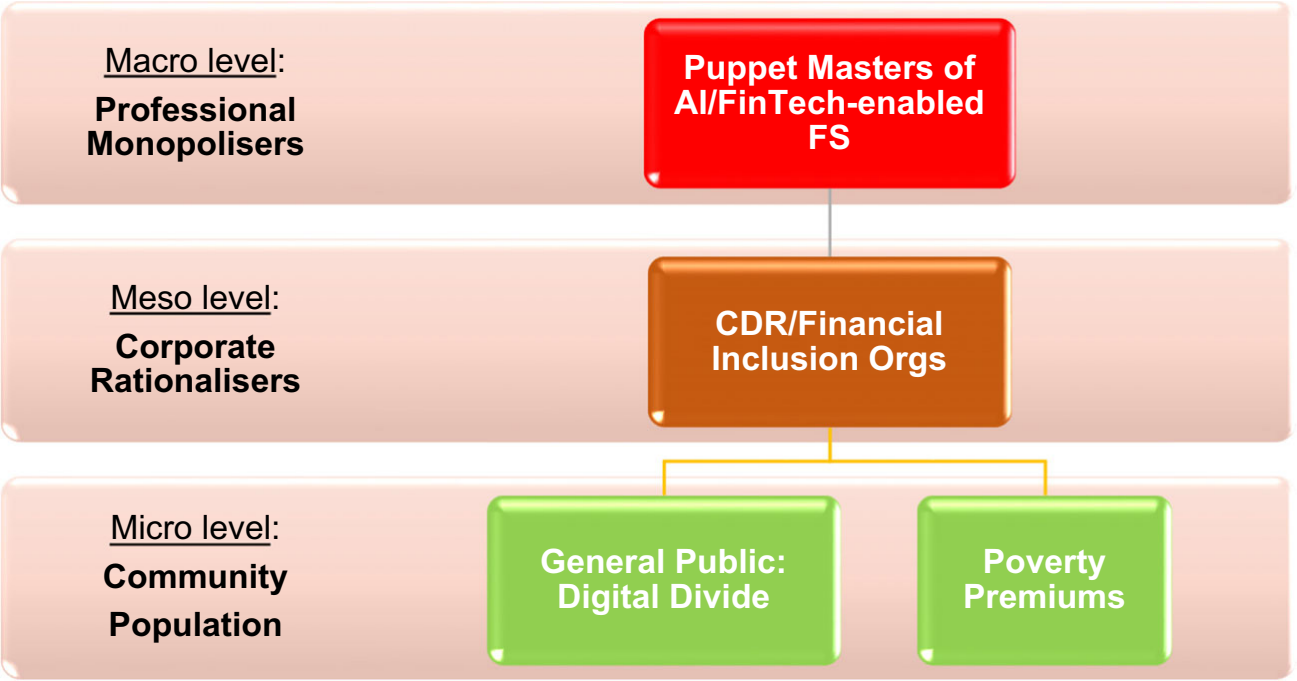
Structured vested interests in the digital society (cf. Alford 1975)

Zuboff’s findings exemplify conflicting interests between the *puppet master* orchestrating the *uncontract dystopia*, reifying Alford’s (1975: xiii) notion of “strategically structured interest” first applied to examine the conflicting interests in health system reforms. Alford contended there existed “a continuing struggle between major structural interests operating within the context of a market society – “professional monopolists” controlling the major . . . resources, “corporate rationalisers” challenging their power, and the community population seeking better . . . care” (ibid. : xiv). Based on the above, we interpret the current digital society in Fig. 1.

Figure 1 echoes Zuboff’s (2019) insights. The monopolist position is the *shadow text* level. The invitation of the monopolisers to the community population to participate in the digital society amounts to “social control”—the *puppet* (Alford, and R.,, and Friedland, R. 1975: 472) or Zuboff’s (2019: 334-6) “blankness of perpetual compliance”. Hence, analogies between Zuboff’s appeal to maintain human qualities of dialogue, problem-solving, and empathy against the “uncontract” environment are juxtaposed against a perceived ineffectual meeting of non-dominant needs and subsequent “low-levels of engagement”, particularly amongst “lower-income individuals” linking to digital inequity of the “community population” residing in the digital divide (ibid.; van Dijk, 2020). We return to discuss the corporate rationalisers or *forward text* role, later in this paper. Alford’s theory has been criticised for not defining which interests are specifically “repressed” by the monopolisers (North and Peckham 2001; Checkland et al. 2009). Nonetheless, the approach is useful and continues to be applied across finance, and to COVID-19, relative to conflicting interests (Ülgen 2017; Briggs 2021). We have acknowledged conflicting interests residing in the digital society, binding us to the “normative or ethical domain . . . Normative issues are intertwined with our very understanding of complexity” (Cilliers 2005: 259-264). In order to make responsible decisions for AI/FinTech-enabled FS and society, we must respect “otherness and difference as values in themselves” (Cilliers 1998: 139). To echo Kunneman (2010: 132), addressing “complexity is not only the central scientific, but also the central *ethical* problem of our time”. We know that algorithms can reconstruct people’s data and discriminate around gender and semantics (Perez 2019; Toreini et al. 2020). However, the subjective concept of “trustworthiness” to remove bias is difficult in computer science engineering discourse and practice; hence, we consider the complexities in defining and building trust, in light of the plethora of ethical principles in circulation.

## Trust, Ethics and Human Oversight of AI

**Fig. 2:**
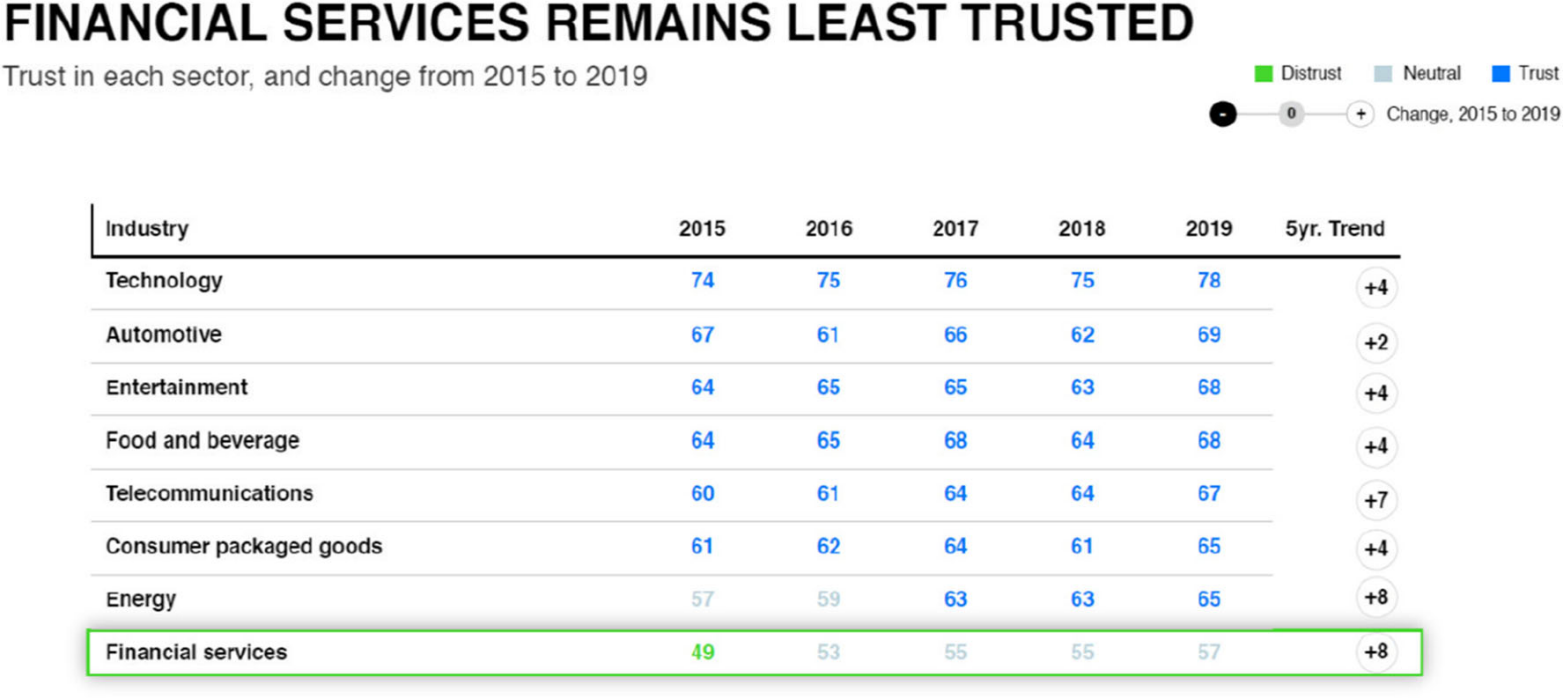
Trust in financial services 2015–2019 (source: Edelman 2019b: 8)

Koshiyama et al. (2021: 2) recognise a series of sociotechnical and ethical issues characterised by the shift from “Big Data” to “Big Algo” in the “5 V” model: (i) *volume*, as resources and know-how proliferate, soon there will be billions of algorithms; (ii) *velocity*, algorithms making real-time decisions with minimal human intervention; (iii) *variety*, from autonomous vehicles to medical treatment, employment, finance, etc.; (iv) *veracity*, reliability, legality, fairness, accuracy, and regulatory compliance as critical features; and (v) *value*, new services, sources of revenue, cost-savings, and industries will be established. Thus, exposing the complexities of definition, debates, opaqueness and what the future holds, nobody has a complete grasp on the full potential of machine learning and AI not to mention its oversight (Hauer 2018, 2019). On the one hand, COVID-19 highlights the increased rate of digital skills, adoption, and transformation across digital society, viewed as a positive outcome of the pandemic (“volume”; Iivari et al. 2020). On the other hand, this trend fails to include the marginalised in society languishing in the digital divide and beholden to the *puppet-puppet master* scenario (van Dijk 2019). Thus, we wrestle with the consequences: when the algorithm is “responsible” for a societal harm, does this align with our societal “veracity” (Abbott 2018; Shaw 2021). Bryson (2021) likens the widespread adoption of AI to “electricity . . .[or] nuclear technology” where variants of the “good” versus “bad” debate weigh heavily on our normative ethical and moral instincts—to ignore the bad in favour of the good aspects of AI would be foolhardy given Zuboff’s (2019) insights. Likewise, we cannot ignore vested interests in advocating and luring society into technological adoption (“variety” and “value”). Mitchell (2019: 145) warns, “the science of electricity was well understood before it was widely commercialized. We are good at predicting the behaviour of electricity . . . [such is] not the case for many of today’s AI-systems” “velocity”. If we want AI/FinTech-enabled FS to be trusted, combined with overcoming the above issues and ramifications post-GFC (2007-9), work is required to re-build trust, given this sector’s current “least trusted” label awarded by its customers (Edelman 2019b; see Fig. 2).

AI’s impact for an equitable digital society is not inevitable (Pasquale 2020). Progress towards equity must be intentional, coordinated, and, crucially, *collaborativ*e. Trustworthy outcomes in AI/FinTech-enabled FS require institutions and organisations to take a holistic approach to the management and oversight of not just financial products, but AI (and the data that empowers it) to engender trust. Trust is not reliant on the AI, or the outcomes it produces; rather, trust in an organisation’s ability to create responsible AI. Despite many traditional financial institutions possessing a social licence to operate, consumer trust is fluid, must be earned, maintained, and as we saw with the GFC (2007-9; Table 1) can easily be lost (Dietz and Gillespie 2012; Aitken et al. 2020).

TRUST requires transparency, responsibility, understanding, stewardship, and truth (Shaw 2020: 176, italics and brackets added):

*Transparency* being clear about what it is you are doing and why . . . the opportunities and risks. . . *Responsibility* being reputable and accountable for what it is you do; *Understanding* . . . you provide [customer] services to understand what outcome they can expect and how it will impact them…and society; *Stewardship…*a good custodian of the data . . in line with the kind of society we all want to create; *Truth,* validating the accuracy of data, the insight and inferences made . . . are beneficial and not harmful.

TRUST must be codified into practical ethical data and AI governance tools, which are not mutually exclusive but understood within an overlapping framework (i.e. AI governance), if we are to innovate for socially beneficial AI/FinTech-enabled FS, worthy of our shared digital futures. Earlier, we referred to the Draft EU AI Regulation (DEAR) published on 21st April 2021.^2^ It delineates a risk-based approach to AI, imposing organisational-level governance in respect of data and quality management procedures and ongoing monitoring, including transparency and provision of information for users^3^ across the AI lifecycle, only vis-a-vis “High-risk AI.”^4^ DEAR is only applicable to the EU-centric AI marketplace, rendering FinTechs outside the EU at a disadvantage, suggesting a lower bar for trust, vis-a-vis EU comparators who want to operate globally. Although DEAR progresses towards providing regulatory impetus for AI governance, giving organisations legitimacy to ascribe budget and costs (i.e. ethical implementation requirements), the guidance permits high-risk AI operators to decide the method/technical solution to achieve compliance. DEAR implementation will also be challenging for EU market-entrant FinTech start-ups compared to larger established competitors, as DEAR leans heavily on governance, market monitoring, and enforcement roles. We argue that embedding TRUST becomes a pivotal method of competitive advantage (Arnold et al. 2019).

Similarly, DEAR affirms the significant void for “cuspy”,^5^ medium- and low-risk AI, devoid of regulatory guidance and practical tools to implement effective AI governance, reliant on voluntary industry, or organisational codes of conduct^6^ to embed them. Thus, AI governance remains in a vacuum, open to the five unethical risks: (1) ethics shopping; (2) ethics blue-washing; (3) ethics lobbying; (4) ethics dumping; and (5) ethics shirking, undermining the potential to trust the use of AI (see Floridi 2019). To engender trust, DEAR calls for standards, conformity assessment, and certification, an end-user facing Conformité Européene (CE) Marking, and registration with a public EU Database for High-Risk AI. However, precise administrative details for compliance remain unclear. DEAR could have collaborated with existing ethical AI standards with shared objectives in progressing to maturity of in-organisation operational processes. For example, the Institute of Electrical and Electronics Engineers (IEEE)^7^ and International Organization for Standardization (ISO)^8^ provide “AI standardization . . . to establish trust in AI-systems”, specifically, an AI playbook^9^ for financial services “to prioritise human well-being and ethical considerations.” While DEAR acknowledges “[a] comprehensive ex-ante conformity assessment through internal checks, combined with a strong ex-post enforcement, could be an effective [solution]”^10^ to promote public trust, DEAR also recognises the nascent status of such processes, including that “expertise for [AI] auditing is only now being accumulated” (ibid.). For instance, ForHumanity (2021)^11^ lead this area in creating an “infrastructure of trust”, through AI-auditing standards and tools that have the potential to impact humans in the areas of “Ethics, Bias, Privacy, Trust and Cybersecurity”. We can infer that these ex ante and ex post governance tools are intended to operate in providing transparency while engaging a “soft-law” normative constraint on “the “do’s” and “don’ts” of algorithmic use in society” (Morley et al. 2020); without enforceability or liability, these “soft law” tools lack “teeth” (Shaw 2021),

Nonetheless, DEAR marks a significant improvement for AI governance, affording organisations the opportunity to find and create risk management, governance, and oversight solutions provided conformity is achieved. Conversely, DEAR risks invoking a “tick-box” compliance culture (FCA 2016; van Vuuren 2020), rather than espousing a digital ethics culture across the AI lifecycle and digital society (see CDR below). This does not compensate for the lack of real and meaningful sociotechnical interaction between internal and external “end user” stakeholders. DEAR underplays the importance of co-governance in stakeholder engagement to de-risk AI and hold it accountable to build trust (Ackerman 2004), in respect of bias, ethical and societal impacts, which ultimately lead to legal consequences for AI systems businesses (Coeckelbergh 2020; Toreini et al. 2020). Such core elements are the heart of outcome-based risks associated with AI systems, which can and do undermine trust. Pivotally, “external ethical auditing will be a key component of any form of operationalised AI-ethics” (Morley et al. 2021: 11), where “individuals have a right to expect the technology they use to perform in a reasonable manner and to respect their trust”^12^. Without trust, social responsibility and operationalising digital ethics through AI-governance, regulation will fail as “consumers won’t use the firm’s services, adopt new technology, or share their data” (Shaw 2020: 176). DEAR provides insufficient methods of “how” to be digitally responsible for AI/FinTech-enabled FS; hence, collective groups are co-creating mechanisms to address responsibility.^13^

## Corporate Digital Responsibility (CDR)

**Fig. 3:**
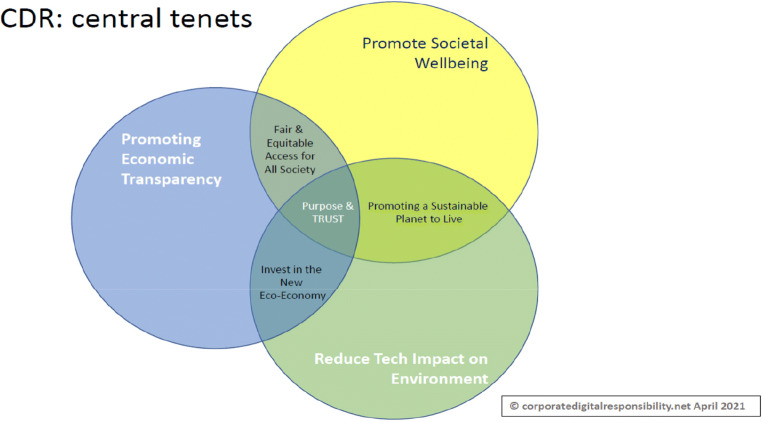
Corporate Digital Responsibility/Digital Responsibility Code (DRC) copyright© CDR.net (https://corporatedigitalresponsibility.co.uk/—draft version, CDR group working on developing this graphic.)

CDR is a voluntary commitment by organisations fulfilling the *corporate rationalisers’* role in representing community interests to inform “good” digital corporate actions and digital sustainability (i.e. data and algorithms) via collaborative guidance on addressing social, economic, and ecological impacts on digital society. For AI/FinTech-enabled FS, the CDR is a potential framework to assist navigating AI governance complexity and to devise an informed strategy. In short, AI governance post-GFC must ensure equity beyond the monopoliser’s interests to include all stakeholders invested in an organisation’s modus operandi (Bell 2020; Pedersen 2021). CDR codifies TRUST and illustrates how AI governance and expectations are met building on lessons learned from corporate social responsibility including environmental and sustainable goals (CSR, Carroll 1991). Limited literature exists, but we advocate CDR as a separate proposition for organisations specifically linked to digital technology and data (Lobschat et al. 2021), not an extension of CSR (Herden et al. 2021). Rather, CDR complements such protocols, as the digital realisation of the same responsibilities but with a two-fold appropriate use of digital and data *within* and *by* the organisation to impact society while sustaining our planet to improve organisational environmental and social governance performance (Dörr 2020). Thus, the combination of environmental and social responsibilities is viewed as the transparent measurement of effectiveness in CDR execution, while accessible for stakeholders to evaluate organisational performance operating within digital society (Bell 2020: 201), illustrated in Fig. 3.

Interpreting Fig. 3 for AI/FinTech-enabled FS governance, “purpose and trust” are the central drivers for the appropriate use of digital and data *within* and *by* the organisation(s). Trust is subjective; therefore, a “declaration of purpose” by organisations is advocated, committing to upholding the collective interests of society and the planet via innovative, appropriate, and permissible use of technologies (Dietz and Den Hartog 2006; Dietz and Gillespie 2012). For example, adhering to a *Digital Responsibility Code* (*DRC*), linked to the notion of green finance, aligned with Sustainable Development Goals (SDGs) across AI lifecycles (Lindenberg 2014, Taghizadeh-Hesarya and Yoshinob 2019). Data efficiency is driven in reducing consumption and measuring emissions using digital technologies to offset carbon impact while investing in environmental, ecotech, and cleantech digital solutions. Likewise, there is a need to embrace tenets of “Fairer access for All” to allay societal fears of AI automation and job loss for governance to succeed. Hence, “good” uses of AI and the economic benefits of digitisation (such as taxation) should be evident to the public (Pasquale 2020). Similarly, responsible engagement with the gig economy (where appropriate) is a must, and abiding by legal, regulatory, and ethical principles per geography and market, and embodying an “open” data approach to demonstrate commitment to the DRC (Shaw 2020, 2021). In so doing, an AI/FinTech-enabled FS can embrace the tenets of CDR while respecting the need for upholding data ownership rights, privacy and the right of an individual to monetise their own data (i.e. open banking).

**Fig. 4:**
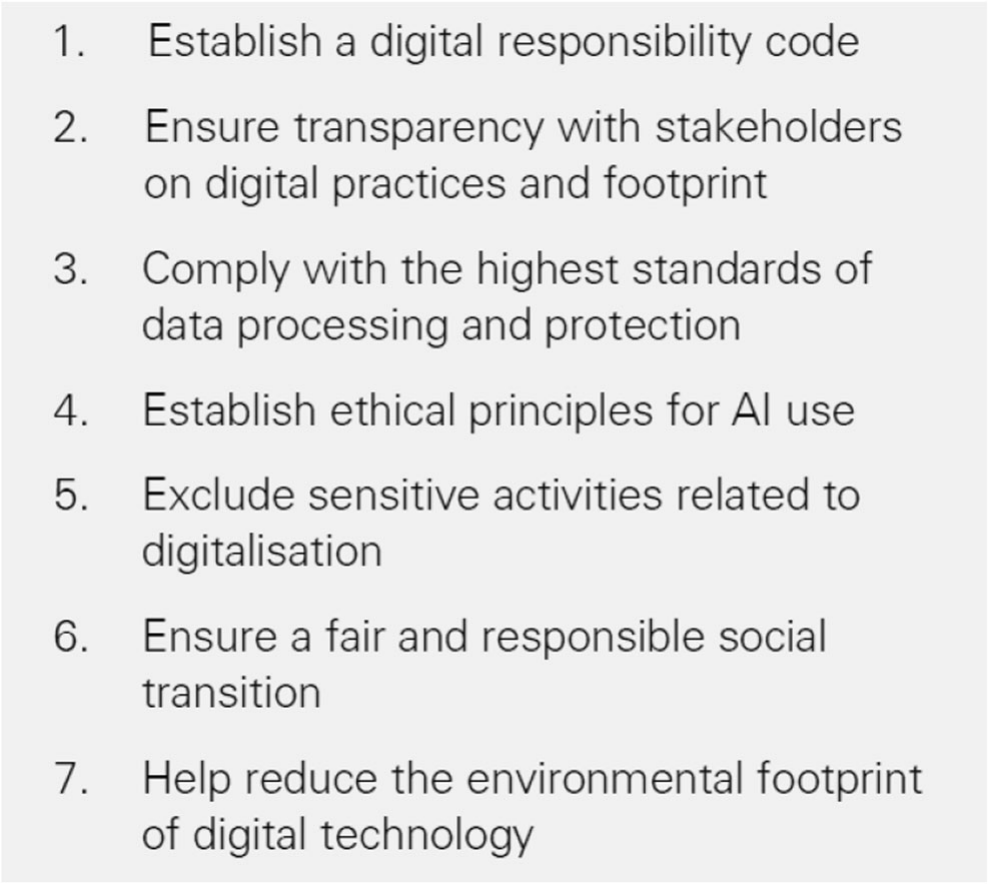
Ethos (2020) “A Method of Digital Responsibility”

Thus, striking a balance between the oversight of digital ethics “within” AI/FinTech-enabled FS in reducing the use of unbiased AI decision-making algorithms (where possible) and opening access to digital technologies through facilitating connectivity, skills, and tools relative to digital finance. In addition, “by” the organisational commitment to cultivating societal digital maturity (understanding), leading to empowered choice, decision-making and well-being (mental health) including the teams across the AI-lifecycle, therefore, embedding purpose and trust to drive AI governance adoption (Wade 2020; Coeckelbergh 2020). Hence, engaging in responsible recycling practices reinforces the DRC via reappropriation of digital devices and promoting the circular economy (Geng et al. 2019) through schools and marginalised areas with collaborative initiatives—the Learning Foundation’s Digital Access For All (DAFA)^14^ offsetting power consumption with renewables for offices and sustainable IT strategies. Finally, the CDR model prescribes appointing a Digital Ethics Council/CDR Advisory Board^15^. We argue that all companies require this aspect of CDR whether that be digital, data or AI focused, or all three. Crucially, this role will and must be complementary to, and of the code, as a dynamic iterative AI governance framework in which the DRC operates and extends responsibility for digital, data or AI services once launched into digital society. Of course, there is a caveat that CDR is not the only collaborative framework and is voluntary, a potential flaw we observed in implementing the DEAR and challenging for smaller AI/FinTech-enabled FS to achieve, but councils and boards can be shared while Dörr’s (2020) text offers practical CDR implementation. CDR creates cultural change to avoid invoking “tick-box” compliance. Early adopters are the Swiss organisation, Ethos, (Fig. 4) demonstrating consideration of the CDR’s tenets, and we witness similar implementation across Germany, France, the UK, the USA, China, and South Korea.^16^

## Our Digital Futures

Society mistrusts AI systems, yet gradually we have succumbed to accept algorithms making potentially life-changing decisions as a daily “norm”. Specifically, within financial services, mistrust is higher post-GFC, where customers remain cautious of this sector’s interests combined with the power afforded by AI/FinTech-enabled FS. We have focused on “what” needs to be considered in governing AI systems and building trust—risk reduction of the potential harms that technologies inflict on our digital society, whereby the underpinning science is not fully understood. Who is responsible to ensure our digital safety as more AI systems are free to operate devoid of human oversight? Responsibility is much debated within the moral and philosophical literature; we have framed the normative aspect and complexity underpinning AI science and its associated ethical principles. Despite a plethora of ethical principles and guidance including the recent DEAR, precisely “how” organisations frame actions and governance and build a digital ethics culture over today’s tendency to opt for the path of least resistance via the tick-box mentality remains challenging. One potential method of the “how” is “Corporate Digital Responsibility” combining the appropriate use of digital and data *within* and *by* the organisation impacting across the social-societal, economic, and environmental systems. Although voluntary for organisations, CDR members draw from their collaborations across standardisation networks centring on better AI-enabled systems (IEEE, iTechlaw and ForHumanity), enabling the equitable digital society to come to fruition. However, collaboration is key, while recognising humans’ dark side in resisting conformity (Klotz and Neubaum 2016); the complexity between our normative and symbiotic selves will continue to be fluid and unpredictable, reflecting AI/FinTech-enabled FS adoption. We must meet this challenge to redress the equilibrium of interests between the current perceived puppet—puppet-master space and avoid an uncontract dystopia. CDR, we posit, can potentially differentiate organisations, facilitating the gaining and maintaining of stakeholder trust and driving competitive advantage.

## Footnotes

1 https://digital-strategy.ec.europa.eu/en/policies/european-approach-artificial-intelligence

2 https://eur-lex.europa.eu/procedure/FI/2021_106

3 “Users” defined in DRAFT Article 3(4) as “‘user’ means any natural or legal person, public authority, agency or other body using an AI system under its authority, except where the AI system is used in the course of a personal non-professional activity”, not end users.

4 Draft Art 6 and Annex III, DRAFT EU AI Regulation supra.

5 AI-systems, on the cusp of the high-risk AI bracket, open to judicial/administrative interpretation.

6 DRAFT Article 69.

7 https://ethicsinaction.ieee.org/p7000/

8 https://www.iso.org/committee/6794475.html

9 https://ethicsinaction.ieee.org/#series

10 Draft Art 6 and Annex III, DRAFT EU AI Regulation supra.

11 US-based, non-profit organisation, https://forhumanity.center/independent-audit-of-ai-systems.

12 https://www.europarl.europa.eu/doceo/document/TA-9-2020-0275_EN.html

13 https://www.itechlaw.org/ResponsibleAI2021

14 See https://digitalaccessforall.co.uk/

15 Interchangeable term

16 See https://corporatedigitalresponsibility.co.uk/links
